# Interobserver reliability in the 3D facial scan assessment of the outcome in dermal filler therapy

**DOI:** 10.1186/s13005-025-00562-1

**Published:** 2025-11-29

**Authors:** Anna Vadasz, Mate Vlocsko, Tamas Tarjanyi, Ferenc Rarosi, Tamas Vereb, Anna Lili Zicsi-Liess, Laszlo Hegedus, Veronika Agnes Jancsik, Jozsef Piffko

**Affiliations:** University of Szeged, Szeged, Hungary

**Keywords:** Orofacial clefts, Dermal filler, 3D facial scanning, Interobserver reliability

## Abstract

**Study design:**

We conducted a prospective cohort study comparing interobserver reliability of 2 different 3D scanning measurement methods following dermal filler therapy in oral cleft patients.

**Objective of the study:**

The objective of the study was to compare the interobserver reliability of 2 different assessment methods of 3D facial scan data.

**Methods:**

We studied 10 oral-cleft patients prospectively who underwent dermal filler therapy in the oral region by a single oral-surgeon using a modified injecting technique with 27 gauge 25 mm micro-cannula between 2021 and 2023. 3D facial scans were made before the treatment procedure and during follow-up visits and were used for analysis. The interbody fusion was scored on these 3D images using the 2 classification systems (Method 1, Method 2) by 3 experienced orthodontic specialists all of whom were blinded to clinical data and outcome.

**Results:**

Of the 2 classifications included in the current study, all classifications had a high interobserver agreement.

**Conclusion:**

The 2 assessment methods used in this study showed high interobserver reliability so we can conclude that, the applied methods may provide as a validation in the outcome of dermal filler therapy. However, we suggest that further refinements may be required to achieve a reliable and consistent assessment of 3D facial scan analysis after dermal filler therapy in oral cleft patients.

## Introduction

A crucial aspect in the outcome of clinical research lies in data obtained from observational measures. Especially when developing new therapeutic approaches, the outcomes must be reliable and reproducible.

In this study, we present a safe and effective technique for lip augmentation with hyaluronic acid in patients with cleft lip scars.

The overall aim of this study was to understand how we can positively impact the lives of individuals with cleft lip scars and evaluate the dynamic outcomes of hyaluronic acid filler therapy.

**Fig. 1:**
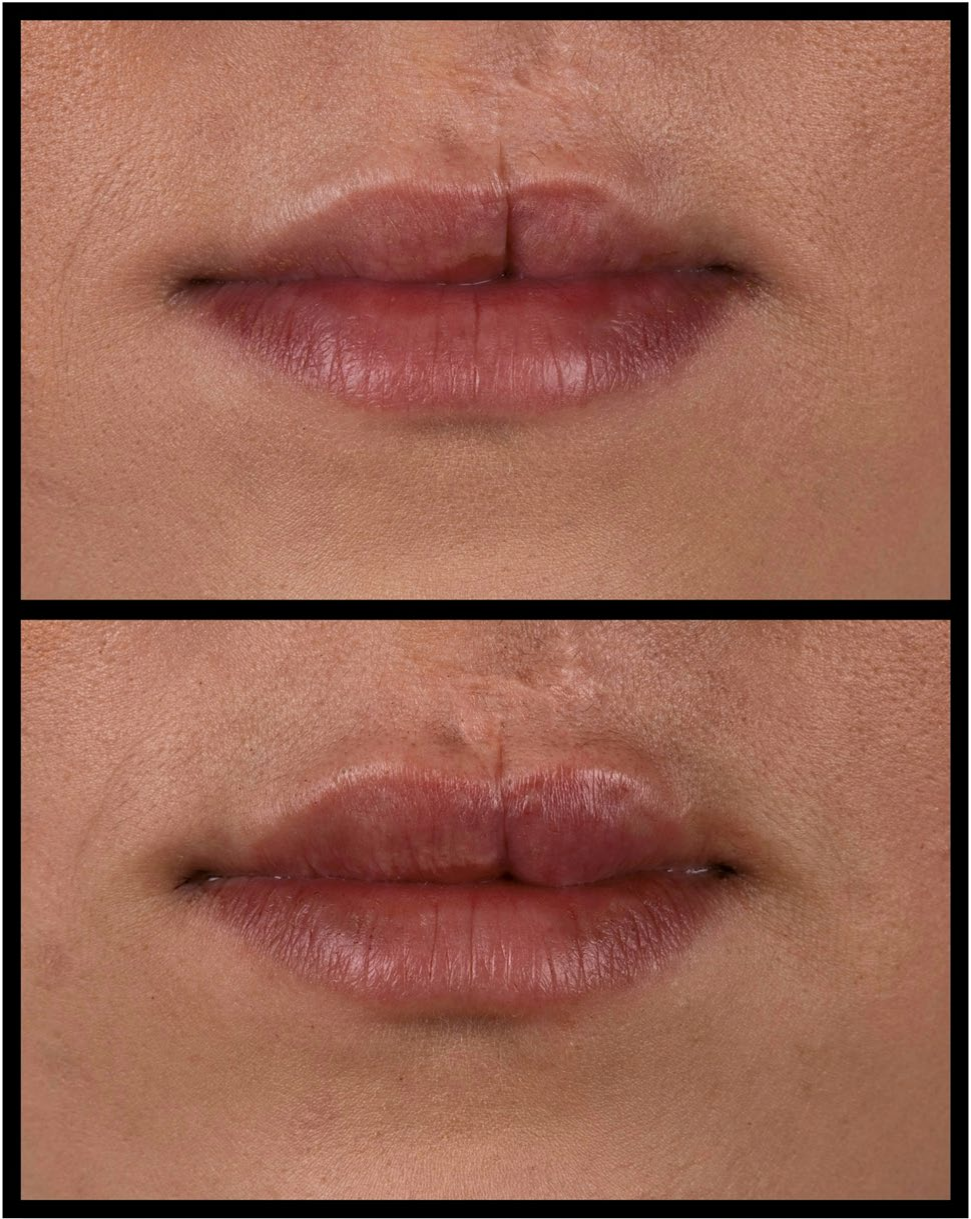
Preoperative and postoperative photo of lip filler therapy

**Fig. 2:**
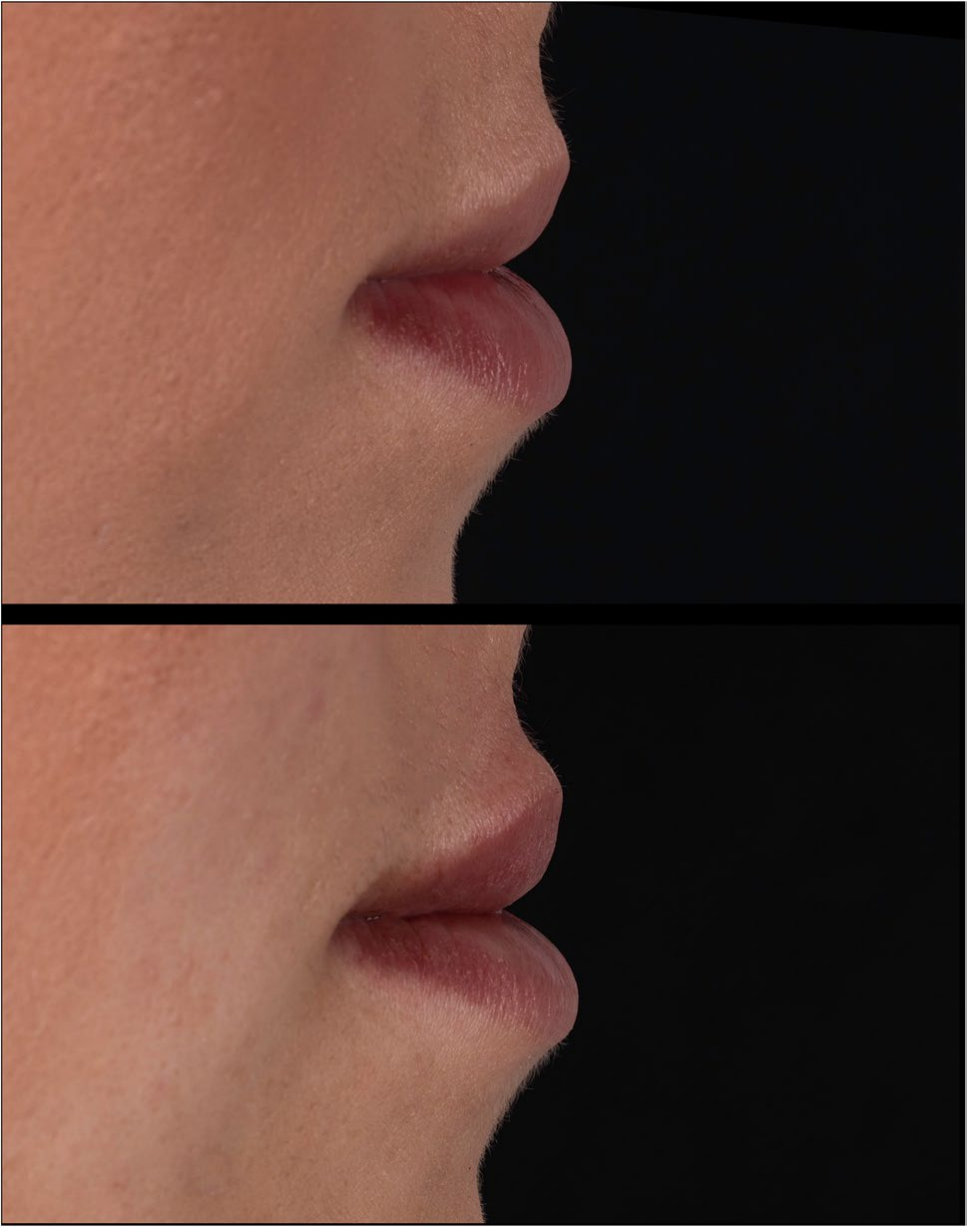
Preoperative and postoperative photo of lip filler therapy

During the last decades, the number of dermal filler procedures performed has increased exponentially. The importance of reliable measurement systems is becoming more and more evident [1], not only to objectively quantify therapeutic results, but also to record patient satisfactory data comparing the accomplished quantitative results [2]. Therefore, several classifications have been developed that use plain photographs of the perioral region to assess the degree of the therapeutic outcome [3]. However, reliable photographic assessment of the outcome after the therapy on 2D photographs remaining to be difficult in this population. These classification systems were developed solely based on the use of digital photography (Figs. 1 and 2) [4]. Even though in current times 3D facial scanning imaging is increasingly used in the assessment of dermal filler therapy, recent recommendations indicate the use of photographic analysis [5]. The purpose of this study was therefore to determine the interobserver of 2 different methods, using static 3D facial scanning from patients who underwent dermal filler procedure with use of hyaluronic acid performed by a single oral surgeon (J.V.A) in our institution. To our knowledge, no studies have been performed to compare these measurement methods in a single cohort of oral cleft patients.

## Materials and methods

### Study design and population

A prospective cohort study was performed of 10 consecutive patients who underwent dermal filler procedure with the use of hyaluronic acid injection for the correction and harmonization of both uni- and bilateral upper cleft lip after surgical correction. All patients were treated by a single oral surgeon between 2021 and 2023. This study was approved by the Hungarian Medical Ethics Committee.

#### Clinical trial number

Not applicable.

### Imaging protocols of the facial scans

3D facial images using the 3D handheld structured-light scanner (Artec EvaTM; Artec Group, Luxembourg) were at the first visit prior to the injection (T0), at the first visit after the injection (T1), one month post intervention (T2), 3 months postoperative (T3), 6 months postoperative (T4) and 12 months postoperative (T5). This scanner uses structured light technology, projecting a grid pattern onto the object to capture precise geometric data with up to 16 frames per second [6, 7]. The scanner boasts a 3D point accuracy of up to 0.1 mm and a resolution of 0.2 mm [7–10], ensuring high-detail capture of even the most intricate surfaces [11]. The frames are adjusted automatically and real-time allowing for the operator to see the 3D model as it is being constructed. All images were taken with the head in a natural position, closed eyes, teeth within centric occlusion and lips in rest [12]. To accomplish the natural head balance, patients were seated in a back-supported and a vertically adjustable chair. They were instructed to turn their heads forward and backward with decreasing amplitude until a relaxed position is achieved [13]; then, they were requested to look straight ahead to the point on the wall in front of them at the eye level [12].

Two types of measurements (Method 1, Method 2) were made using the facial scans.

#### Method 1 – frontal view

**Fig. 3:**
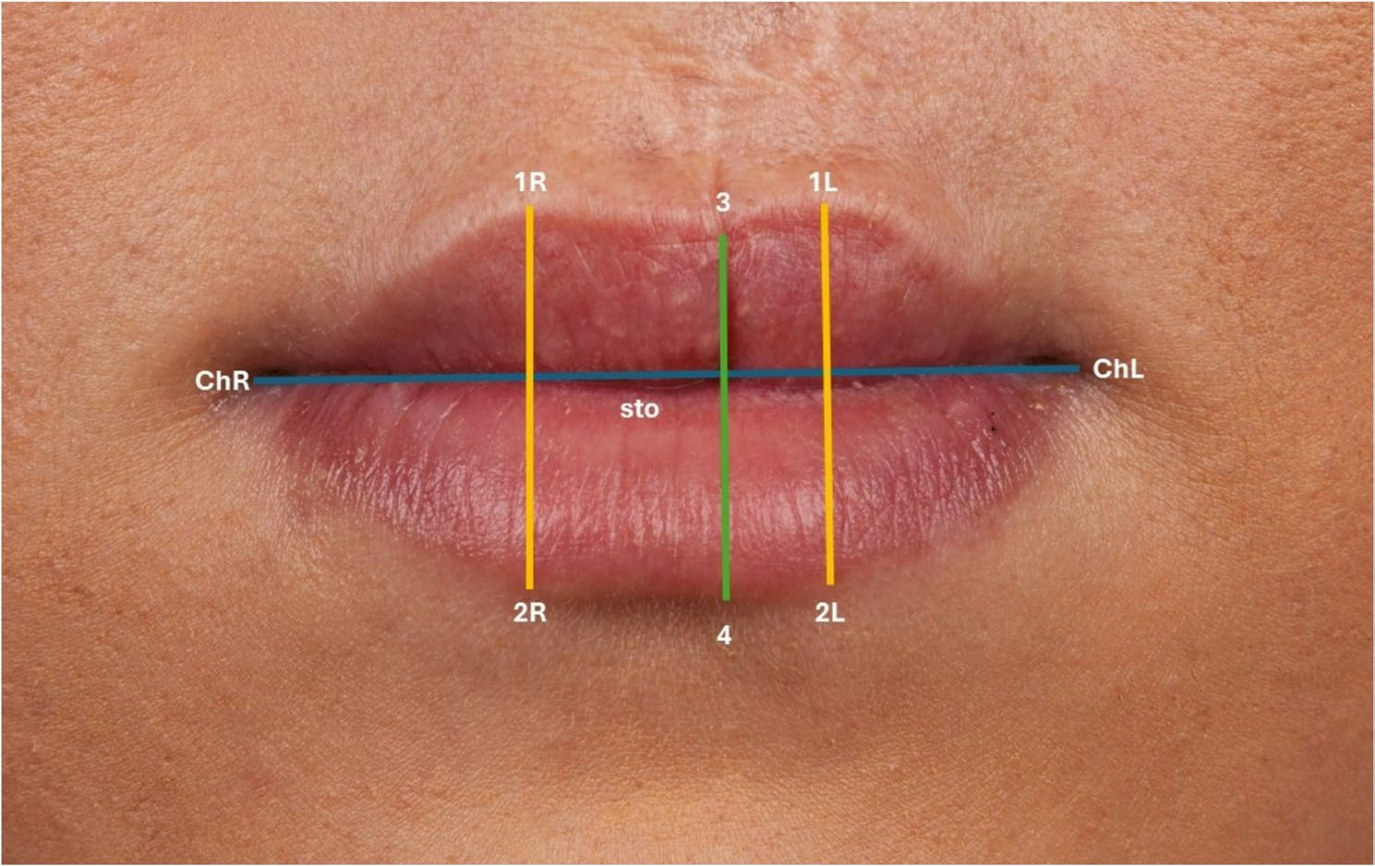
Landmarks of the lip in the frontal view

Eleven landmarks, 8 bilateral and 3 unilateral, were located according to the literature [14, 15] (Fig. 3). Ten linear measurements were taken directly with the 3D-facial images using Artec Studio V.15 software.

Linear distances (mm)

1. mouth width (chR – chL).
2. total red lip height median (3–4)
3. upper red lip height median (3 – sto).
4. lower red lip height median (sto-4).
5. total red lip height lateral-right (1R-2R).
6. upper red lip height lateral-right (1R – sto).
7. lower red lip height lateral-right (sto-2R).
8. total red lip height lateral-left (1–2 L).
9. upper red lip height lateral-left (1 L – sto).
10. lower red lip height lateral-left (sto-2 L).

Standardization the 3D face scan position for reproducibility and comparability.

**Fig. 4:**
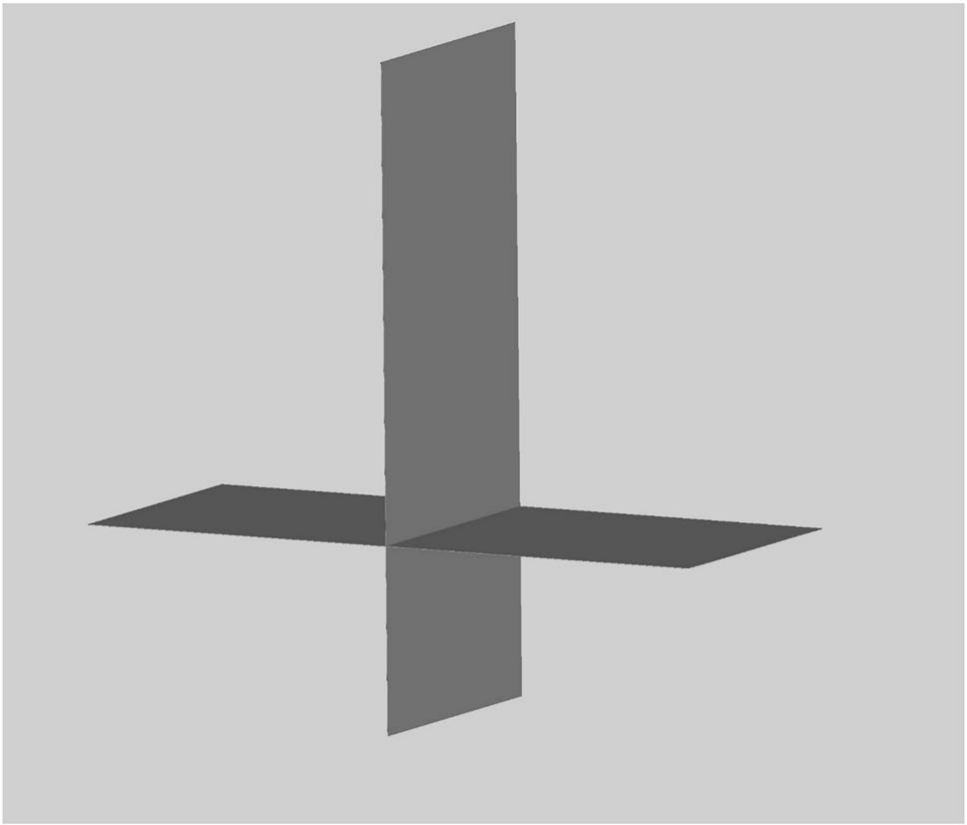
Sagittal and horizontal plane for the 3D face image orientation in Autodesk Meshmixer software

**Fig. 5:**
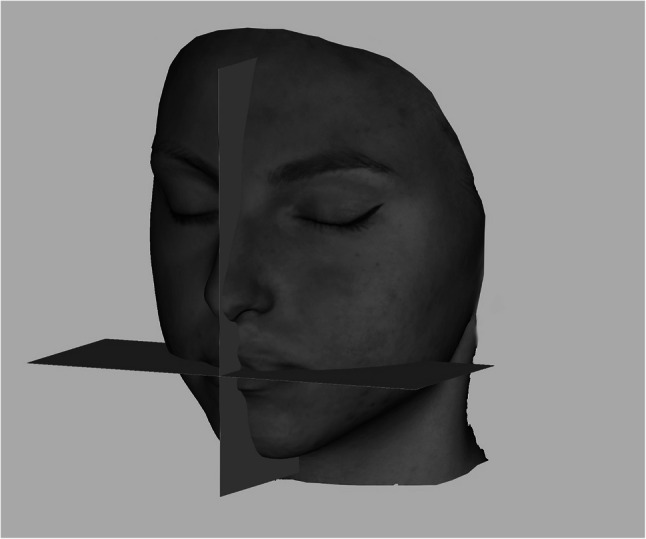
Positioning the 3D face scan due to the sagittal and horizontal planes in Artec Eva software

**Fig. 6:**
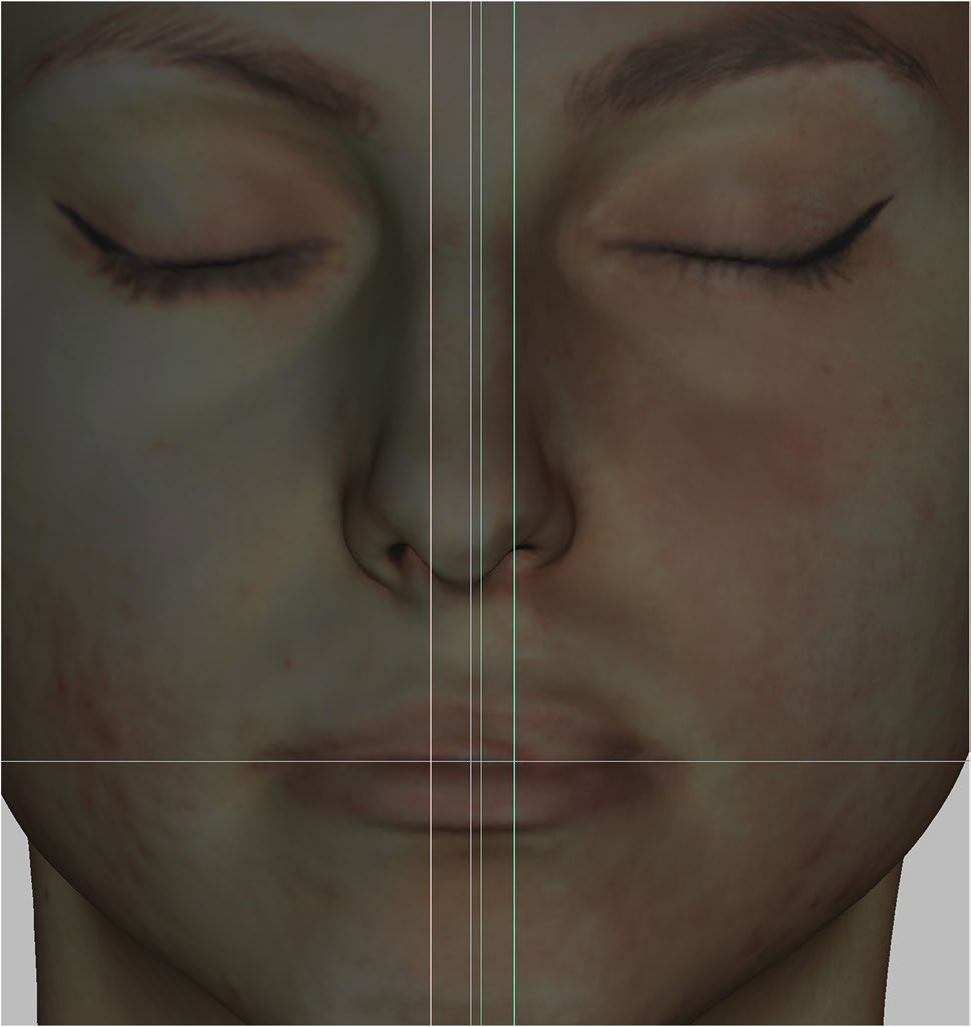
3 parallel planes were created by duplicating the sagittal plane in Artec Eva software

**Fig. 7:**
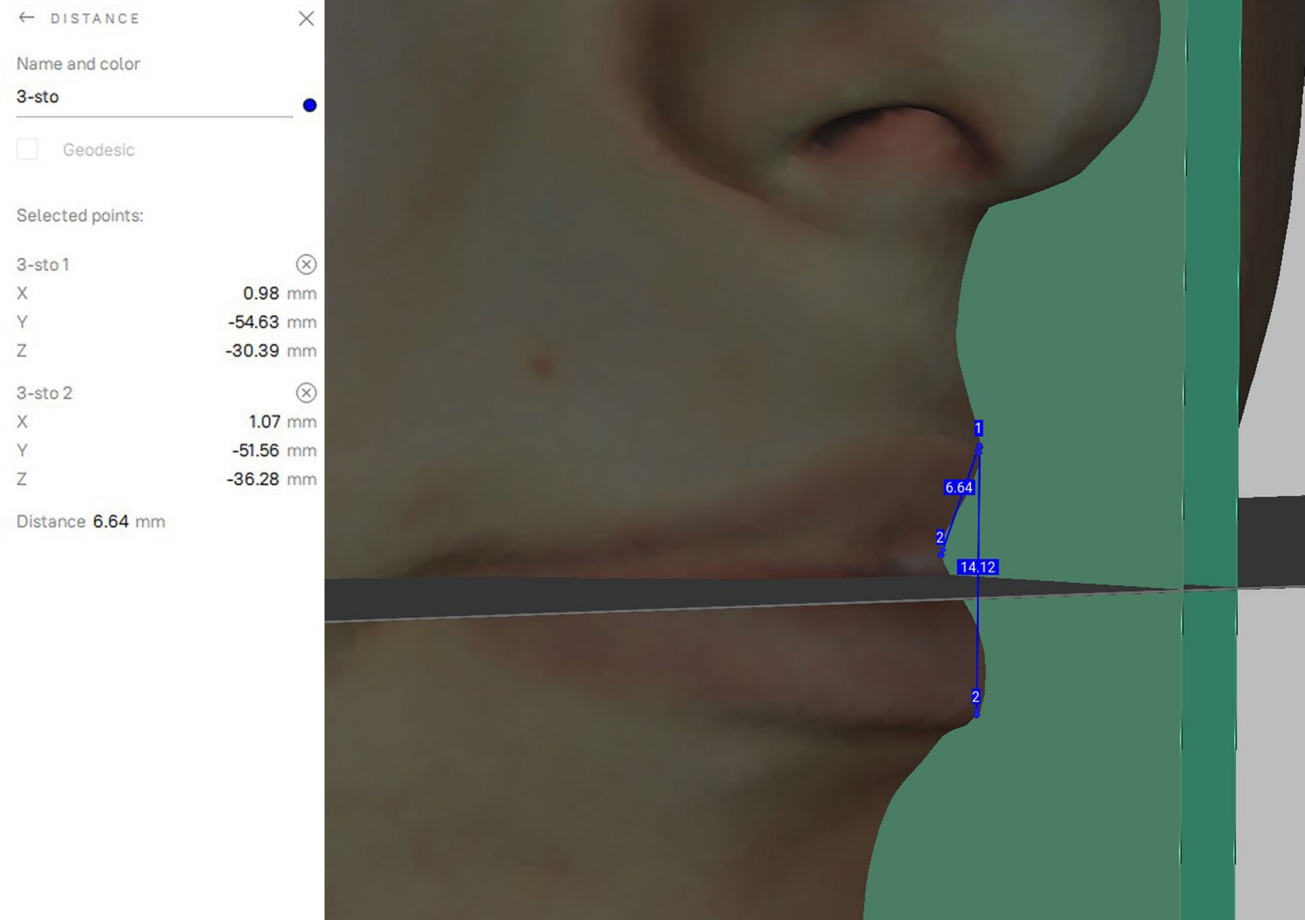
Measuring linear distances between the landmarks of the lip along the planes in Artec Eva sofware

Considering that the software places the images in a random location in it’s coordinate system, a method had to be invented to always measure the images in the same plane and position. The 3D face images had to be positioned in the same way and direction during the measurements to compare the data [8]. 2 planes, a sagittal and a horizontal (perpendicular to the sagittal plane) STL files were made using open-source Autodesk Meshmixer software (Fig. 4). Importing the colored sproj 3D images into the Artec Studio Software this two planes were also added. The horizontal and sagittal plane were locked so they were not movable. The facial scans were moved in the right direction using the planes (eyes towards the frontal view, scalp to the top) while the sagittal plane went through the facial midline [15] and the horizontal went through the stomion (Fig. 5). Then the 3D head image was locked and 3 parallel planes were created by duplicating the sagittal plane. The first plane went through the most superior point of the Cupid’s bow on the right (1R), the second plane went through the most inferior point of the Cupid’s bow (3) and the third plane went through the Cupid’s bow on the left (1 L). All these 3 planes were perpendicular to the horizontal plane as well (Fig. 6). The section of the first plane on the lower red lip border on the right was point 2R, the section of the second plane on the lower red lip border was point 4 and the section of the third plane on the lower red lip border on the left was point 2 L. Linear distances (mm) were measured between these points in every patient at T0, T1, T2, T3, T4, T5 (Fig. 7).

For comparability the T0 scans with the planes were exported together into one STL file so the further scans could be aligned to it with Artec-Eva Auto-alignment and the anatomical distances could be measured along the same planes. First the T1 sproj file was imported and then the T0 STL and the two planes (sagittal and horizontal). The T0 STL was locked so it was not movable and with the auto-alignment the T1 sproj file aligned to the STL and moved to the same, right direction. The sproj file was also locked, and after the original sagittal plane was duplicated 3 times, they were moved until they covered perfectly the planes of the STL and were fitted perfectly.

According to the interobserver variability all scans were measured by 3 different operators. Every operator set the initial standard head positions of the patients, putting the sagittal plane due to the facial midline, considering the soft tissue nasion, midpoint of the intercanthal distance and symmetry based on individual observation. The other panes were also placed according to the anatomical points.

#### Method 2 – lateral view

**Fig. 8:**
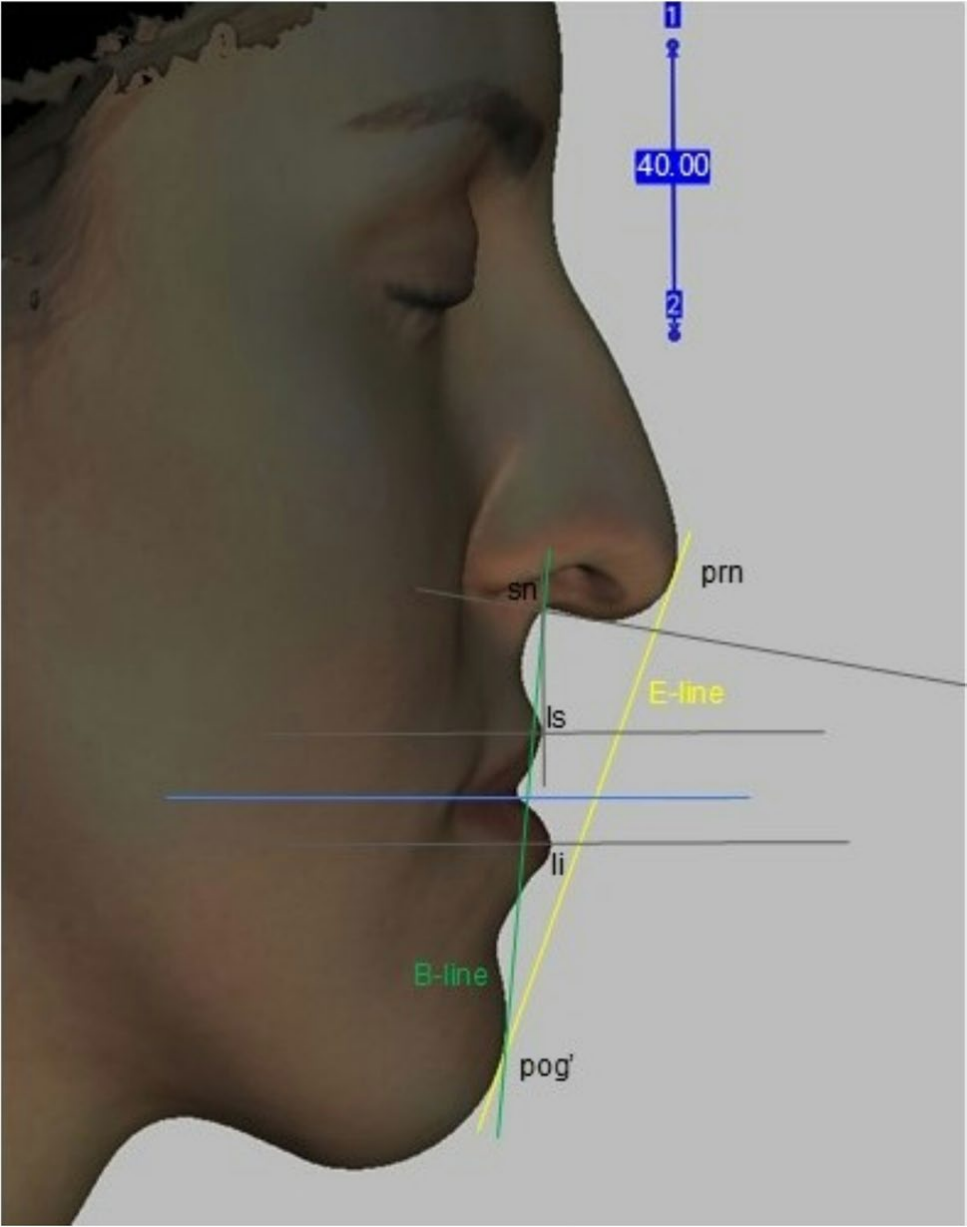
Lateral view of a 3D facial scan with soft tissue landmarks in Artec Eva software

The positioned 3D facial images were turned to the left for a lateral orthogonal point of view like cephalometry. The right side of the patient was seen with the planes. Only the horizontal and midsagittal plane were made visible so the head position could be set totally lateral. In these conditions the horizontal plane looked like only one line. A measuring scale was created in Artec Eva software on the surface of the midsagittal plane, the visibility of the plane was turned off, so the whole contour of the face was visible. For all subjects the following soft tissue landmarks, lines and angle were placed: pronasale (prn), subnasale (sn), labiale superius (ls), labiale inferius (li), soft tissue pogonion (pog’), E-line, B-line, nasolabial angle (Fig. 8).

Lip enhancement was evaluated using 2D image analysis. Lip projection was assessed by measuring the distance (mm) from a reference line to the most protruding point of the upper and lower lip vermillion border (on a line parallel to the original horizontal plane). The reference line was Burstone’s line (B-line). An ad hoc analysis was conducted using Ricketts’ line (E-line) as a reference point to assess upper lip projection.

Cephalometric landmarks and their definition [15]:

- Pronasale: the most prominent point of the tip of the nose in the midline.
- Subnasale: midpoint of the columella base at the columella-labial junction. The point at which the columella merges with the upper lip in the midsagittal plane.
- Labiale superius: the most anterior midline point on the upper lip in the sagittal plane.
- Labiale inferius: the most anterior midline point on the lower lip in the sagittal plane.
- Soft tissue pogonion: the most prominent point on the soft tissue chin.
- Ricketts” E-line (E-line): the line from the pronasale to the soft tissue pogonion.
- Burstone’s line (B-line): the line drawn from the soft tissue pogonion to the subnasale.
- Nasolabial angle: the intersection of two lines, one drawn from the base of the nose (subnasale) to the tip of the nose and another extending from the subnasale to the most anterior point of the upper lip (labiale superius).

### Statistical analysis

#### Study population

A total of 10 patients were included after performing dermal filler therapy. Patient characteristics are described in Table 1.

**Table 1:** Patient characteristics

	*N* = 10
Age at follow-up (y)	23 (20–28)
Age at injection (y)	22 (19–27)
Duration of the follow-up (y)	1
Sex [ n (%)]	
Female	6 (60)
Male	4 (40)
Type of cleft [ n (%)]	
Unilateral	6 (60)
Bilateral	4 (40)

### Interobserver variation

#### Method

To compare the measuring person the repeated measure analysis of variance was performed using a general linear model (GLM) for each measured variable with six levels as the different time points, with parwise comparisons by using Sidak post hoc test. The significance level was set as 0.05.

## Result

The repeated measure ANOVA showed no significant differences in the mean values between the measuring persons for all variables (Table 2).

**Table 2:** Statistical analysis for interobserver reproducibility

v_1R_2R	Measuring person 1	Measureing person 2	Measuring person 3
Measuring person 1	-	0.858	0.798
Measuring person 2		-	0.999
Measuring person 3			-

v_1R_sto	Measuring person 1	Measuring person 2	Measuring person 3
Measuring person 1	-	0.828	0.745
Measuring person 2		-	0.999
Measuring person 3			-

v_sto_2R	Measuring person 1	Measuring person 2	Measuring person 3
Measuring person 1	-	1.000	0.997
Measuring person 2		-	0.999
Measuring person 3			-

v_1L_2L	Measuring person 1	Measuring person 2	Measuring person 3
Measuring person 1	-	0.922	0.836
Measuring person 2		-	0.997
Measuring person 3			-

v_1L_sto	Measuring person 1	Measuring person 2	Measuring person 3
Measuring person 1	-	0.913	0.868
Measuring person 2		-	0.999
Measuring person 3			-

v_sto_2L	Measuring person 1	Measuring person 2	Measuring person 3
Measuring person 1	-	1.000	1.000
Measuring person 2		-	1.000
Measuring person 3			-

v_3_4	Measuring person 1	Measuring person 2	Measuring person 3
Measuring person 1	-	0.860	0.827
Measuring person 2		-	1.000
Measuring person 3			-

v_3_sto	Measuring person 1	Measuring person 2	Measuring person 3
Measuring person 1	-	0.650	0.649
Measuring person 2		-	1.000
Measuring person 3			-

v_sto4	Measuring person 1	Measuring person 2	Measuring person 3
Measuring person 1	-	1.000	0.993
Measuring person 2		-	0.994
Measuring person 3			-

## Discussion

The treatment of clefts in the orofacial region requires a multiteam collaboration, but this topic is remaining as a challenging task in the field of oral and maxillofacial surgery. The surgical correction of lip asymmetries has limitations: due to lack of the amount of the tissues, differences in the growth patterns of the tissues of the lips, the risk of scar building in the philtral area [16]. However, asymmetries of the face and lip has become more popular and are indicators of someone’s uniqueness [17] in our time, patients suffering from scars in the lip area are mostly unhappy with their appearance [2].

In clinical practice, classification systems are often used in assessing prognosis of disease, communication and treatment strategies [17, 18]. A reliable treatment option should be replicated by different researchers while producing the same results. Unfortunately, 3D facial scan analysing system may have limits in its reproducibility [19]. Inter-rater agreement for 3D analysis is essential to practicing physicians. Inter-observational reliability measures the degree of agreement between different professionals assessing the same data. In this prospective cohort study we investigated the inter-observer reliability of two different measurement 3D facial scanning methods. The frontal (Method 1) and lateral (Method 2) view revealed a high interobserver agreement. Although the 2D photographical assessment of the injected area still remains the gold standard to verify the results of the outcome of treatment [3–5]. Therefore, to assess the therapeutic success after dermal filler therapy in a reliable and consistent manner, the applied 2 methods require further investigation. Currently, there is still a need for a reliable classification system, aiding in standardized treatment and outcome of 3D facial analysis in oral cleft patients.

## Conclusion

In this study we investigated the interobserver reliability of 2 measurement methods assessing the therapeutic outcomes of dermal filler therapy in oral cleft patient. There are no gold-standard methods available for analysing 3D facial scans for the assessment of dermal filler therapy for orofacial clefts. The 2 assessment methods used in this study showed high interobserver reliability so we can conclude that the applied methods may provide as a validation in the outcome of dermal filler therapy. However, we suggest that further refinements may be required to achieve a reliable and consistent assessment of 3D facial scan analysis after dermal filler therapy in oral cleft patients.

## Data Availability

No datasets were generated or analysed during the current study.
